# Mitochondrial heteroplasmic shifts reveal a positive selection of breast cancer

**DOI:** 10.1186/s12967-023-04534-4

**Published:** 2023-10-05

**Authors:** Yanni Li, Kristina Sundquist, Sakshi Vats, Mun-Gwan Hong, Xiao Wang, Yilun Chen, Anna Hedelius, Lao H. Saal, Jan Sundquist, Ashfaque A. Memon

**Affiliations:** 1 Center for Primary Health Care Research, Lund University/Region Skåne, Malmö, Sweden; 2 Department of Family Medicine and Community Health, Department of Population Health Science and Policy, Icahn School of Medicine at Mount Sinai, New York, NY USA; 3 Department of Functional Pathology, Center for Community-Based Healthcare Research and Education (CoHRE), School of Medicine, Shimane University, Matsue, Japan; 4 Science for Life Laboratory, Department of Biochemistry and Biophysics, National Bioinformatics Infrastructure Sweden, Stockholm University, Solna, Sweden; 5 Division of Oncology, Department of Clinical Sciences Lund, Lund University, Medicon Village, Lund, Sweden; 6 Center for Primary Health Care Research Wallenberg Laboratory, Skåne University Hospital, 5th floor, Inga Marie Nilssons gata 53, S-205 02 Malmö, Sweden

**Keywords:** Breast cancer, mtDNA, Sequencing, Heteroplasmic mutation, ddPCR validation

## Abstract

**Background:**

Breast cancer is, despite screening, not always detected early enough and is together with other tumor types known to shed genetic information in circulation. Unlike single-copy nuclear DNA, mitochondrial DNA (mtDNA) copies range from 100s to 10,000s per cell, thus providing a potentially alternative to identify potential missing cancer information in circulation at an early stage.

**Methods:**

To characterize mitochondrial mutation landscapes in breast cancer, whole mtDNA sequencing and bioinformatics analyses were performed on 86 breast cancer biopsies and 50 available matched baseline cancer-free whole blood samples from the same individuals, selected from a cohort of middle-aged women in Sweden. To determine whether the mutations can be detected in blood plasma prior to cancer diagnosis, we further designed a nested case-control study (n = 663) and validated the shortlisted mutations using droplet digital PCR.

**Results:**

We detected different mutation landscapes between biopsies and matched whole blood samples. Compared to whole blood samples, mtDNA from biopsies had higher heteroplasmic mutations in the D-loop region (*P* = 0.02), *RNR2* (*P* = 0.005), *COX1* (*P* = 0.037) and *CYTB* (*P* = 0.006). Furthermore, the germline mtDNA mutations had higher heteroplasmy level than the lost (*P* = 0.002) and de novo mutations (*P* = 0.04). The nonsynonymous to synonymous substitution ratio (dN/dS) was higher for the heteroplasmic mutations (*P* = 7.25 × 10^−12^) than that for the homoplasmic mutations, but the de novo (*P* = 0.06) and lost mutations (*P* = 0.03) had lower dN/dS than the germline mutations. Interestingly, we found that the critical regions for mitochondrial transcription: MT-HSP1 (odds ratio [OR]: 21.41), MT-TFH (OR: 7.70) and MT-TAS2 (OR: 3.62), had significantly higher heteroplasmic mutations than the rest of the D-loop sub-regions. Finally, we found that the presence of mt.16093T > C mutation increases 67% risk of developing breast cancer.

**Conclusions:**

Our findings show that mitochondrial genetic landscape changes during cancer pathogenesis and positive selection of mtDNA heteroplasmic mutations in breast cancer. Most importantly, the mitochondrial mutations identified in biopsies can be traced back in matched plasma samples and could potentially be used as early breast cancer diagnostic biomarkers.

**Supplementary Information:**

The online version contains supplementary material available at 10.1186/s12967-023-04534-4.

## Introduction

Breast cancer arises in the terminal duct lobular unit of the breast and is the most common malignancy worldwide, which alone accounts for 30% of female cancers [1]. The common risk factors for breast cancer are age, childbearing, breastfeeding, mammographic density, overweight and obesity, physical inactivity, alcohol consumption and unhealthy lifestyle [2]. Breast cancer is a heterogeneous disease at the molecular level, with a significant genetic predisposition, often associated with mutations in genes such as *BRCA1*, *BRCA2*, *TP53*, *PTEN*, *CDH1*, *STK11*, *CHEK2* and *PALB2*, however, there is still missing genetic information which needs to be identified [3]. Most patients with breast cancer diagnosed at an early stage are curable; patients with advanced-stage or distant metastases are considered incurable using currently available therapies [4]. Imaging-based clinical methods have been used for the early detection and clinical staging of breast cancer. Earlier clinical detection of breast cancer could dramatically improve the survival rate of patients [5]. It is now well-established that molecular changes appear earlier than the clinical diagnosis and identification of such molecular changes may further improve an early diagnosis and prognosis of breast cancer. Cancer sheds information into the blood circulation during tumorigenesis (liquid biopsy), thus providing a great opportunity to detect cancer in the blood long before it can be detected by the prevalent clinical mammography. Cancer cells exploit existing cellular machinery mediated by nuclear and mitochondria for their survival and propagation [6]. Both PCR and NGS-based methods are continuously being developed to detect circulating tumor DNA for early breast cancer detection. However, a feasible detection is difficult due to the few circulating copies of nuclear genomes in the blood at an early stage of cancer [7]. Mitochondrial DNA (mtDNA) may therefore provide an excellent opportunity for early detection because, unlike the nuclear genome, it has two to four orders of magnitude more copies per cell and hence, a greater abundance in circulation.

The mitochondrion is the essential organelle for all types of cells to produce energy, as well as being central to the physiology of humans, including cell signaling, metabolism, apoptosis, and calcium homeostasis [8]. Mitochondrion has its own genome, which is strictly maternally inherited and poses a gene-dense structure with only 16,569 base pairs (bp) but encoded 13 mitochondrial proteins, 22 tRNAs, and 2 rRNAs [9]. Certain types of mtDNA mutations are implicated in severe lifelong mitochondrial-related metabolic diseases [10]. However, the importance of mtDNA mutations in cancers was only recently defined owing to technological development, with several large-scale studies showing the significance of circulating mtDNA in a variety of cancers [11, 12]. MtDNA mutations occur more frequently compared to nuclear DNA (about 100-fold higher), where the median mutation rate is between 0.5 and 10 per megabase (Mb) depending on tumor type [13]. The mtDNA mutation has been regarded as a major source of driver mutations in cancer [14]. Researchers have explored mtDNA content (copy number) or mutations as biomarkers with prognostic and predictive value in breast cancer [15–17]. Recently, a study has shown that mtDNA mutations identified in breast cancer biopsies can also be identified in matched peripheral blood collected just before surgical treatment from the same patients [18]. They further showed that it is not the presence of mtDNA mutation(s) in itself, but the number of mutations vs. normal copies (heteroplasmy) that plays a significant role in cancer pathogenesis [18]. Therefore, in the present study, we first aimed to characterize the mutational landscapes of mtDNA from Swedish population-based breast cancer patients, in their tumor biopsy tissues as well as their matched baseline whole blood samples obtained prior to cancer diagnosis, to identify the de novo mtDNA mutations. Key identified mutations were validated and screened in plasma samples collected prior to the clinical diagnosis of cancer, and the predictive value of these mutations was investigated in a nested case-control study.

## Materials and methods

### Study population

This study was conducted using data derived from the Women’s Health in Lund Area (WHILA) cohort, which is a prospective Swedish population-based cohort that started in 1995. All women, aged from 50 to 59 years old (born between 1935 and 1945) and living in southern Sweden, were invited to participate in the health survey following written informed consent and without financial compensation. From December 1995 to February 2000, 6917 women out of approximately 10,766 (the total eligible women in the five southern municipalities in Scania, 1995) underwent a physical examination and answered a questionnaire [19]. All participants were followed from the day of screening until death or until no event occurred by 31 May 2015, whichever came first. The plasma samples were collected from all participants when they were recruited (baseline). However, the whole blood samples began to be collected after half of the women had already been recruited (October 1997), and therefore 3225 participants with baseline whole blood samples were included in the present study.

For mtDNA sequencing, after excluding prevalent cancers, a total of 349 women (~ 10%) were diagnosed with breast cancer during follow-up. 345 of these women also had baseline plasma samples collected when they were cancer-free. 173 women had matched diagnostic biopsies and we included only those subjects who were diagnosed with breast cancer within 3 years of inclusion, as we did not anticipate detecting mutations before that period. Based on these criteria, we identified 86 tumor biopsies, and where available, we also sequenced the matched baseline whole blood mtDNA samples (n = 50) to investigate germline mutations and identify any de novo somatic mutations.

For droplet digital PCR (ddPCR) validation and detection in plasma samples from patients prior to cancer diagnosis within the WHILA cohort, cell-free DNA (cfDNA) was extracted from available plasma samples of patients (n = 304) diagnosed with breast cancer during follow-up and 359 matched controls (age and date of sampling, Additional file 2: Table S5).

### Outcome measurement

**Fig. 1:**
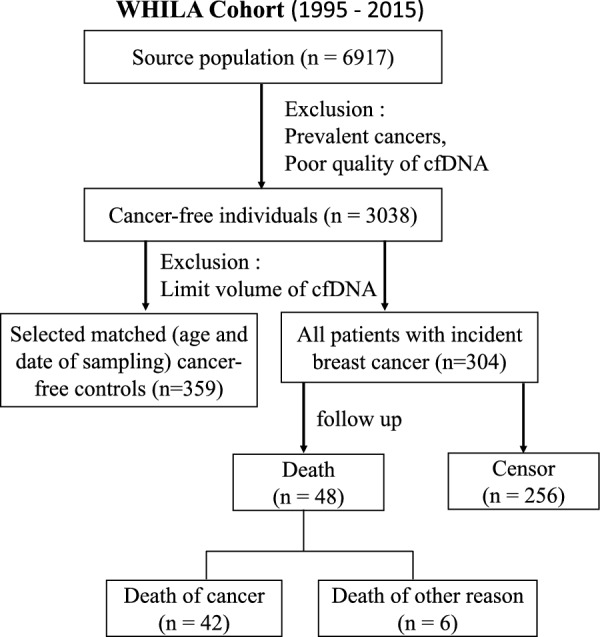
Flow chart of participants in the nested case-control study

We retrieved the information on breast cancer incidences and mortality from the Swedish Cancer Register and the death register, and information on prevalent cancer was obtained from self-reported questionnaires. Of the participants included in the study, 187 had been diagnosed with cancer at baseline (prevalent cancer) and 3038 were cancer-free. We followed cancer-free women from the day of screening until (1) cancer diagnosis; (2) death; (3) the end date of this study (31 May 2015). During an average follow-up of 18.3 years, we identified a total of 304 women with incident breast cancer. To further investigate the relationship between identified mtDNA mutations and mortality rate, we identified 48 patients who died from any cause (overall mortality) and among them, 42 died from cancer (cancer mortality) (see Fig. 1).

### Assessment of covariates

Information on potential confounding factors at baseline was collected through the health survey, including age at screening, BMI, education (1–9, 10–11, ≥ 12 years of schooling), smoking habit (non-smokers, past smokers, current smokers), alcohol habit (no consumption, < 12 g/day, ≥ 12 g/day), physical activity at home with a score between 1 and 3 was defined as low activity at home (1: hardly does anything at all; 2: mostly sedentary; 3: light physical exertion) and a score between 4 and 6 was high activity at home (4: strenuous exercises 1–2 h/week; 5: strenuous exercises at least 3 h/week; 6: hard regular exercise), physical activity at work (low, moderate, high). Information on the prevalent diseases and first-degree family history of cancer (either mother or sister had any type of cancer was included) was collected from the health survey and information on incidence diagnoses of diabetes, hypertension was obtained through nationwide medical registers (the Swedish health registers).

### DNA extraction and library preparation

The biopsy samples were snap-frozen immediately after surgery and stored at – 80 °C in the South Swedish Breast Cancer Group tumor bank. The total DNA from biopsies was extracted from biopsies using the QIAamp Nucleic Acid Kit (Qiagen, Germany) according to the manufacturer’s instructions. The whole blood samples were collected at baseline in standard EDTA blood tubes. Total blood genomic DNA was extracted using QIAamp96 DNA Blood (Qiagen, Germany) from a 200 µL blood sample according to the manufacturer’s instructions. Total cfDNA was extracted from plasma samples using the QIAamp Circulating Nucleic Acid Kit (Qiagen, Germany). The concentrations and purities of isolated DNA samples were spectrometrically analyzed and frozen at − 20 °C for further usage. To verify the extraction efficiency, an internal control (DNA Spike 166 bp, TATAA Biocenter, Sweden) of known concentration was added to each sample and samples with an extraction efficiency of more than 90% were included.

We used two pairs of primers and applied long-range PCR to amplify the whole mitochondrial genome from biopsy and whole blood samples. The sequences of the primers are listed below:

**MTL_Fwd1**: AAAGCACATACCAAGGCCAC

**MTL_Rev1**: TTGGCTCTCCTTGCAAAGTT

**MTL_Fwd2**: TTGGCTCTCCTTGCAAAGTT

**MTL_Rev2**: AATGTTGAGCCGTAGATGCC

Followed by the library preparations using Nextera DNA flex library preparation kit (Illumina, USA) according to the manufacturer’s instructions. The concentrations of the purified libraries were determined using the Qubit 4.0–1X ds DNA high sensitivity assay (Invitrogen, USA) and the size (500-1000 bp) was verified using Experion electrophoresis (Bio-Rad, USA). The final library pool was diluted to 80–100 pM (with 2% internal control, phiX) and was loaded into our in-house iSeq100 system. After the sequencing, we obtained dual-index, paired-end raw fastq files.

### MtDNA variants calling and quality control

We performed quality control on the raw reads using MultiQC v1.8 [20] and removed the adapters using TrimGalore v0.6.5 [21]. The cleaned files were analyzed using multifunctional integrated software -- MToolBox v1.0 with the configurations: mtdb_fasta = chrM.fa, hg19_fasta = hg19RCRS.fa, mtdb = chrM, humandb = hg19RCRS, input_type = fastq, ref = RCRS, UseMarkDuplicates = true, UseIndelRealigner = true and MitoExtraction = false, hf_min = 0.1, hf_max = 0.9, minrd = 5, minqual = 25. During the processing, the Revised Cambridge Reference Sequence (RCRS) served as mitochondrial reference genome. MToolBox employed the mapExome.py to realign the fastq reads with GSNAP, permitting multiple alignments per read within a certain penalty score threshold. Subsequently, the assembleMTgenome.py was ran to reconstruct complete mitochondrial genomes [22]. Following the alignment and reconstruction steps, a Variant Call Format (VCF) file containing the mtDNA mutations including insertions and deletions (ins/dels) and the heteroplasmy level (HL) of each variant allele with the related confidence interval (CI) was generated for each sample. BCFtools v1.8 [23] was used to individually normalize VCFs and merge across all samples. The final VCF was annotated by combining the NCBI Homo sapiens Annotation and the mitochondrial DNA function locations from MITOMAP (https://www.mitomap.org/MITOMAP) [24]. The haplogroup for each mtDNA sequence was determined using the Haplogrep v2.4.0 [25].

After obtaining all the variants, we performed further quality control on the mutations and defined heteroplasmic mutations (the ratio between mutant and wild type) by adopting and modifying the criteria of Wei et al., 2019 [26], as follows: (1) retained the mutations with the lower bound of the CI of heteroplasmic variant allele frequency (VAF) below 1%, (2) discarded mutations at sites with multiple alternate alleles and remove the alleles with the HL less than 5%, (3) re-categorized heteroplasmic mutations at sites with over 98% of the upper bound of the CI as homoplasmic mutation, (4) removal of mutations falling within specific regions associated with misalignment errors related to homopolymeric tracts, (5) removal of heteroplasmic variants with sequencing depth < 100× or low HL (< 5%) with depth < 250×.

### MtDNA mutation validation

For validation, we shortlisted the identified mutations based on the following criteria: (1) heteroplasmic mutation, (2) HL of variant > 15%, (3) mutation frequency in the study population > 5%, (4) the mutation which has high HL in biopsy than the whole blood sample and (5) potential functional mutation. Based on the selection criterion, we shortlisted mt.1888G > A in the *RNR2* gene, which had HL ranging from 63 to 89.3% (~ 7% prevalence in biopsies) and mt.16093T > C presented in the D-loop and it had HL ranged from 14.4 to 97% (~ 8% prevalence in biopsies), for further validation.

### ddPCR validation

ddPCR validation of selected mutations on the baseline plasma cfDNA was performed using the QX200 AutoDG Droplet Digital PCR System (Bio-Rad, USA) according to the manufacturer’s instructions. The mutation assays for mt.1888G > A and mt.16093T > C were designed and all primers, probes and reagents were ordered from BioRad. The probes used to quantify the mutations contained either a 5′-FAM (mutant) or 5′-HEX (wild type) reporter dye and HPLC-purified Iowa black fluorescent quencher. The probes were stored at 4 °C and primer details for all assays can be found below:

**Mt.1888G > A:**

MIQE Context: 4ec16488bb305da9800c1ad1d37d68aa|seq1:140–262:+

CAAGGACTAACCCCTATACCTTCTGCATAATGAATTAACTAGAAATAACTTTGCAAGGAGA[G/A]CCAAAGCTAAGACCCCCGAAACCAGACGAGCTACCTAAGAACAGCTAAAAGAGCACACCCG

**Mt.16093T > C:**

MIQE Context: 4f645a5f181d564bb6a9baaf6fe0b62e|seq1:140–262:+

TGGGGAAGCAGATTTGGGTACCACCCAAGTATTGACTCACCCATCAACAACCGCTATGTAT[T/C]TCGTACATTACTGCCAGCCACCATGAATATTGTACNGTACCATAAATACTTGACCACCTGT

For the amplification, each 22 µL ddPCR reaction contained 11 µL of 2× ddPCR SuperMix for probes (no dUTP), 4 µL template ctDNA (about 2ng in total), 1 µL FAM- and HEX-labelled probes and 1 µL Restriction enzyme HindIII (5U, Thermo Scientific, USA). Reactions were prepared in a semi-skirted 96-well plate (Eppendorf) and sealed with an automated pierceable foil heat sealer. Following droplet generation on the AutoDG, the plate was sealed with the heat sealer and PCR was performed on a T100™ thermal cycler (Bio-Rad) with the parameters as follows: 95 °C for 10 min for enzyme activation, then followed by 40 cycles of 94 °C for 30 s for denaturation and at 54 °C for 1 min for annealing/extension, at 98 °C for 10 min for enzyme deactivation. After PCR, the plate was incubated at 4 °C overnight, which can maximize the droplet recovery. The plate was then read using a QX200 droplet reader (Bio-Rad) and the data were analyzed using QuantaSoft Software (Bio-Rad) to determine the numbers of droplets that contained the positive and negative fluorophore for each sample.

### Statistics

All statistical analyses were carried out using R version 4.1.2 (https://cran.r-project.org/).

For clinical data, the student’s t test was used to compare the continuous variables (age at baseline, BMI) and the Pearson chi-square test was used for categorical variables, such as education level, smoking habits, alcohol consumption, activity at work, activity at home, diabetes, hypertension, obesity, first-degree family history of cancer. The logistic regression analysis was performed to evaluate the association between mt.1888G > A, mt.16093T > C and cancer incidence. Odds ratios (ORs) and 95% confidence intervals (95% CIs) were calculated to evaluate the association between mt.1888G > A, mt.16093T > C and breast cancer risk. The Cox proportional hazards model was performed to evaluate the association between mt.1888G > A, mt.16093T > C and cancer mortality. Hazard ratios (HRs) and 95% confidence intervals (95% CIs) were calculated to evaluate the association between mt.1888G > A, mt.16093T > C and breast cancer mortality.

We performed different tests when comparing mtDNA mutation between biopsies and whole blood samples. Poisson regression and Wilcoxon rank sum test were used to compare the difference between the counts and between the levels, respectively. Fisher’s exact test was performed to compare mutations (signature) in two groups at different mitochondrial regions or mutation consequences. The binomial test was used to compare the difference in the number of negative or positive mitochondrial HL shifts within the same mitochondrial region. Bonferroni method was applied for multiple testing correction.

## Results

### The mtDNA mutation landscape of breast biopsies and matched whole blood samples

The mtDNA from 86 breast cancer biopsies and available matched 50 baseline whole blood samples from the same patients were sequenced to characterize and identify mutations (mean depth in the biopsies = 1105×; mean depth in the whole blood samples = 1218×) (see Additional file 1).

After quality control (QC) steps and filters, in the breast cancer biopsies we identified a total of 1788 homoplasmic mutations at 415 unique mtDNA sites, 735 heteroplasmic mutations at 419 sites and 191 insertions/deletions (InDels) at 51 sites. To understand the mtDNA mutation landscape of biopsies, we investigated the characteristics of these mutations (Table 1 and Additional file 2: Table S1). We observed that each participant had at least 29 mutations including on average 8.5 heteroplasmies and 18.53 homoplasmies, and 2.16 InDels. In contrast to the nuclear genome (transition-to-transversion (Ti/Tv) ratio ~ 2), the Ti/Tv ratio was more than 5 for all mutations across our study population. The higher Ti/Tv ratio shows the robustness of our data and is consistent with mitochondrial polymerase γ base misincorporation error [27, 28] in breast cancers. Here, The mtDNA coding region had more nonsynonymous mutations (dN/dS > 1), indicating that these mutations in the mitochondrial genes were potentially under the positive selection in cancer [29]. The mutation burden was lower in CpG sites of mtDNA in general.

**Table 1:** Mutation characteristics of mtDNA in breast cancer biopsies

Region	Length (bp)	No. of mutations per sample	No. of InDels per sample	Ti/Tv	dN/dS	CpG/Non-CpG
D-loop	1122	6.87	0.36	5.63	–	0.06
Gene	11,341	14.78	1.01	16.29	1.17	0.11
rRNA	2513	4.07	0.78	16.45	–	0.03
tRNA	1504	1.31	0.01	27	–	0.15

Columns are defined as follows: The length is the total number of bases by mitochondrial DNA region. No of mutations/InDels is the total number of mutations divided by sample size. Ti/Tv is the ratio of transition-to-transversion change calculated by the number of mutations with A/G or T/C (transition) change divided by the number of mutations with A/C or T/G (transversion) change. dN/dS is the ratio of nonsynonymous to synonymous substitution calculated by the number of nonsynonymous mutations divided by synonymous mutations in the protein-coding region. CpG/Non-CpG is calculated by the number of mutations presented at CpG sites divided by the number of variants presented at Non-CpG sites.

**Fig. 2:**
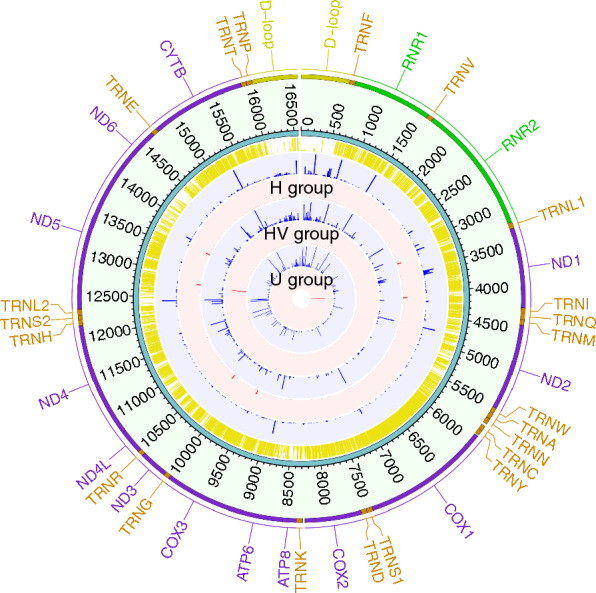
The distribution of mutations and assessment of the pathogenicity score in major haplogroups. Circos plot summarizing all of the mtDNA variants in biopsies. From outside of the circle to inside: (1) genes on the mitochondrial genome, colored by the genome region as yellow: D-loop; purple: coding genes; green: rRNAs; orange: tRNAs. (2) mtDNA position. (3) phastCons100 conservation scores from UCSC (range 0 to 1 from inner to outer ring, where score > 0.5 as conserved sites and < 0.5 as non-conserved sites). (4) heteroplasmy level (HL) of all mutations in H group. (5) HL of disease-causing mutations in H group. (6) HL of all mutations in HV group. (7) HL of disease-causing mutations in HV group. (8) HL of all mutations in U group. (9) HL of disease-causing mutations in U group. From (4– 9), HL from inner 0% to outer 100% (homoplasmic mutation), blue color: HL of all mutations, red color: HL of disease-causing mutations

When stratified by mtDNA haplogroup, haplogroup H (60.5%) was the most frequent in our study population followed by haplogroup HV (17.4%), U (17.4%), JT (2.3%) and M (2.3%). Several mutations were commonly presented within/across the haplogroup background(s). To determine whether those mutations were pathogenic, we compared the mtDNA mutations of different haplogroups with the well-defined disease-causing mutations [30], and 8 out of 57 proposed pathogenic mutations were found in this study (Fig. 2 and Additional file 2: Table S2).

**Fig. 3:**
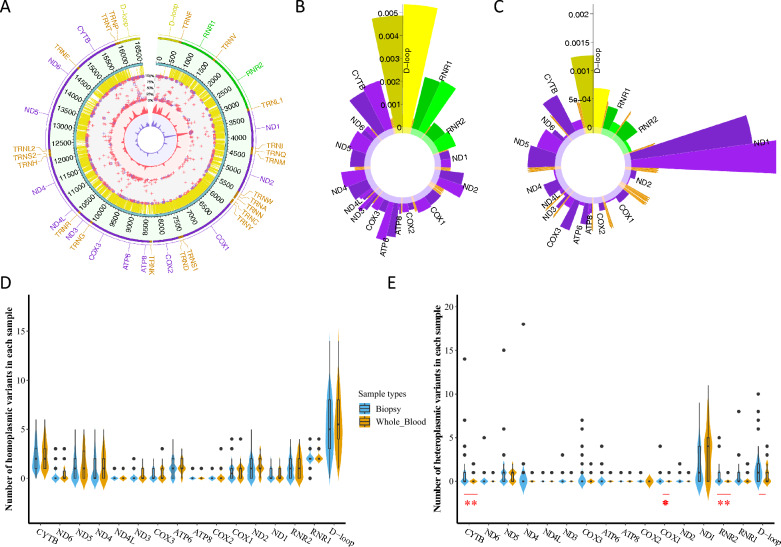
The mtDNA mutation profiles of 86 biopsies and available matched 50 whole blood samples. **A** Circos plot of mitochondrial mutations identified in all biopsies and matched whole blood samples. From outside of the circle to inside: (1) genes on the mitochondrial genome, colored by the genome region as yellow: D-loop; purple: coding genes; green: rRNAs; orange: tRNAs. (2) mtDNA position. (3) phastCons100 conservation scores from UCSC. (4) identified mutations, the radial axis corresponds to the heteroplasmy level from inner 0% to outer 100%, the red cross indicates biopsies, while blues are whole blood samples. The mutations observed in both samples of the same individual are presented with squares. (5) The density of mutations. To make the two tracks comparable, the density was adjusted to reflect the difference in the number of mutations of the two groups. **a** Red: biopsies. **b** Blue: whole blood samples. **B**,** C** The mutation rate of mtDNA genomic regions. The vertical axes represent the number of refined homoplasmic (**B**) or heteroplasmic (**C**) mutations rate per base in each sample. The darker color is biopsies and the lighter color is the whole blood samples. Mitochondrial genes are displayed and colored by the regions as **A**. **D**, **E** Comparison of biopsies and whole blood samples regarding mutations in mitochondrial genes, respective *P* value obtained with Poisson regression. ** = *P* < 0.01, * = *P* < 0.05

In whole blood, from the mtDNA sequencing data, we identified 1172 homoplasmic mutations at 302 unique sites, 315 heteroplasmic mutations at 85 sites and 129 InDels at 25 sites. We found that the majority of mtDNA mutations were common in both biopsies and matched whole blood samples (both homoplasmic and heteroplasmic) (Fig. 3. A–C). We further performed Poisson regression analyses and compared the hetero- and homo-plasmic mutation occurrence in biopsies and whole blood samples, results showed that D-loop (*P* = 0.02), *RNR2* (*P* = 0.005), *COX1* (*P* = 0.037) and *CYTB* (*P* = 0.006) had more heteroplasmic mutations in biopsies than whole blood samples (Fig. 3. C, E). In contrast, the *ND1* gene had a higher heteroplasmic mutation load in the whole blood sample than in the biopsies (*P* = 0.052, Fig. 3. B, D). There was no statistical difference between the homoplasmic mutation frequencies in biopsies and whole blood samples (Fig. 3. B, D), suggesting that those homoplasmic mutations were most likely germline mutations.

### The mtDNA mutation landscape of 50 matched biopsy-whole blood pairs

We performed a subgroup analysis on 50 patients with available matched biopsies and whole blood samples and found similar trends of both hetero- and homo-plasmic mutation rates (Additional file 2: Fig. S1A–D, Fig. 3B–E). Overall, there were no statistical differences regarding hetero- and homo-plasmic mutations and InDels in the mitochondrial functional region between two groups (Additional file 2: Fig. S1E, F). For those 36 biopsies without baseline whole blood samples, we separately examined the mtDNA mutational landscape and found a consistent pattern in mutation occurrence, Ti/Tv ratio, dN/dS ratio, and CpG/Non-CpG ratio across all samples (Additional file 2: Table S3), implying that the subgroup analysis of 50 biopsies with matched baseline samples serves as a representative sample set for the broader study population.

**Fig. 4:**
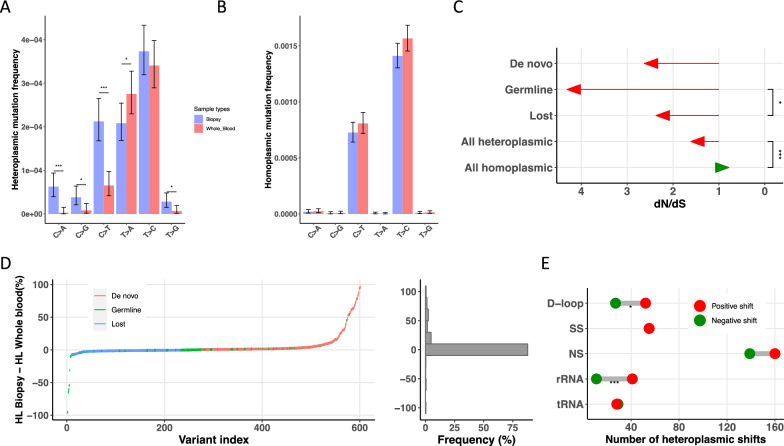
Mitochondrial heteroplasmic shift reveals the positive selection of breast cancer. **A**, **B** Comparison of heteroplasmic (**A**) and homoplasmic (**B**) nucleotide mutational spectra in the mitochondrial genome from the matched 50 pair samples. Statistical significance for the difference (*P*) was calculated using Fisher’s exact test. **C** dN/dS for all types of muatations. *P* was calculated using Fisher’s exact test. **D** Left, difference in the percentage shift of heteroplasmy level (HL) between biopsy and the matched whole blood (HL Biopsy − HL whole blood) ordered by the degree of shift. Right, distribution of the difference of the percentage shift of HL between biopsy and the matched whole blood sample. **E** Number of heteroplasmies showing an increased or decreased HL in each mtDNA region in biopsy-whole blood pairs. *P* was calculated using the binomial test and Bonferroni correction. *P* in Fig. 4 was marked with asterisks: *** = *P* < 0.001, ** = *P* < 0.01, * = *P* < 0.05

Subsequently, we examined the nucleotide hetero- and homo-plasmic mutational spectra in the mitochondrial genome for the 50 matched biopsy-whole blood pairs in detail by performing the Fisher’s exact test. We observed that biopsies have higher C to A (*P* < 0.001), C to G (*P* = 0.01), C to T (*P* < 0.001), and T to G (*P* = 0.02) heteroplasmic substitutions, but lower T to A (*P* = 0.04) substitution as compared to matched whole blood samples (Fig. 4A), which suggests that cancer biopsies may suffer more from spontaneous deamination of cytosine. Indeed, the heteroplasmic mutations in biopsies had very different have very different trinucleotide patterns compared to matched whole blood samples (Additional file 2: Fig. S2A, B). No statistical differences in homoplasmic mutational spectra were observed between the two groups (Fig. 4B, Additional file 2: Fig. S2C, D). Furthermore, to categorize the mitochondrial heteroplasmic mutation in the biopsy-whole blood pairs, we defined the mutations into three categories: (1) germline: the mutation present in both sample types, (2) de novo: the mutation only present in biopsies, and (3) lost: the mutation only present in whole blood samples. Our results showed that the heteroplasmy level (HL) of germline mutations (mean HL: 10.7%, standard deviation [SD]: 23.1%) was higher than the HL of lost mutations (mean HL: 3.8%, SD: 9.6%) in the whole blood samples (*P* = 0.002, Wilcoxon rank sum test), and the HL of germline mutations (mean HL: 14.1%, SD: 24.6%) was higher than that of de novo mutations (mean HL: 9.9%, SD: 20.3%) in the biopsies (*P* = 0.04, Wilcoxon rank sum test). When stratifying the mitochondrial genome into functional regions, we found that nonsynonymous and synonymous mutations had different distributions (*P* = 0.003, Kolmogorov–Smirnoff test). The overall dN/dS was higher for the heteroplasmic mutations than that for the homoplasmic mutations (*P* = 7.25 × 10^−12^, Fisher’s exact test), but the de novo and lost heteroplasmic mutations had lower dN/dS than the germline mutations (de novo vs. germline: *P* = 0.06; lost vs. germline: *P* = 0.03, Fisher’s exact test) (Fig. 4. C). For the de novo mutations that were present only in biopsies, the dN/dS of the major coding genes, except *ATP8* and *ND4L* which have no nonsynonymous variant, were > 1 (Additional file 2: Table S4). However, when compared the detailed heteroplasmic level shift (HL in biopsies – HL in whole blood sample) in three mutation types, the difference between HL in biopsies and matched whole blood samples can be seen in percentages (Fig. 4D). The results showed that both the germline and the lost mutation had a very low level of heteroplasmic shift while the de novo mutations had a high level of heteroplasmic shift, suggesting that it was not the presence of mutations but the heteroplasmy level shift that was important for cancer pathogenesis. Moreover, when characterized the mtDNA into functional region, we observed that mutations in the D-loop (adjusted *P* = 0.03, binomial test) and rRNA genes (adjusted *P* < 0.001, binomial test) had higher HL in biopsies than the matched whole blood samples (Fig. 4.E, Additional file 2: Fig. S1G). These results imply that the breast cancers have has a distinct heteroplasmic mutation signature compared to the baseline whole blood sample from the same individual, suggesting that mitochondrial genetic landscape changes during cancer pathogenesis and the dN/dS and heteroplasmic shift revealed a positive selection in breast cancer.

### The distribution of heteroplasmic variants in the D-loop

**Fig. 5:**
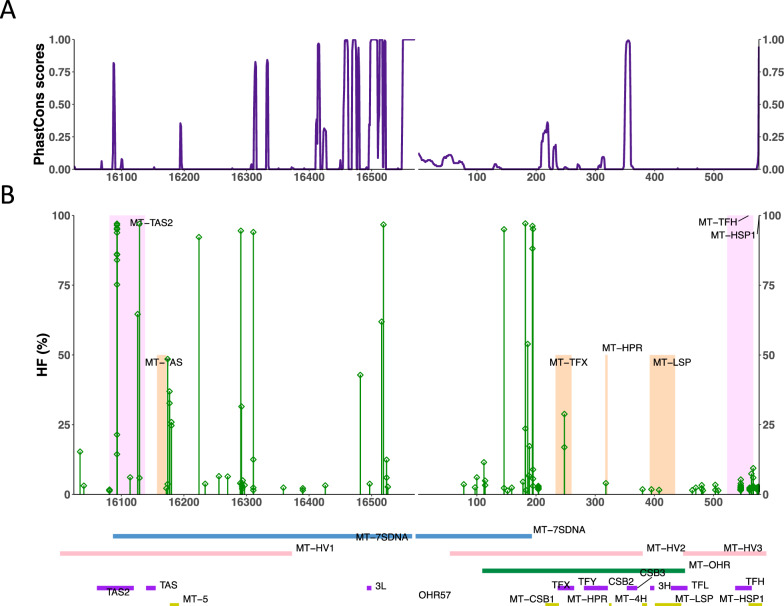
The distribution of mitochondrial heteroplasmic mutations in D-loop. **A** phastCons100 conservation scores across the mtDNA D-loop region (where score > 0.5 as conserved sites and < 0.5 as non-conserved sites). **B** heteroplasmic mutations in the D-loop region. Corresponding known 17 sub-regions of the mtDNA D-loop are shown at the bottom

D-loop is the control region for mtDNA transcription, which can be divided into 17 sub-regions. To explore the possible effects of mutations on this important regulatory region, we analyzed the D-loop mutations in the 50 biopsy-whole blood pairs. We found that 3 of the 17 regions had a significantly higher number of heteroplasmic mutations than the rest of the sub-region in D-loop (Fig. 5 A, B): MT-HSP1 (adjusted *P* = 6.22 × 10^−22^, odds ratio (OR) = 21.41), MT-TFH (adjusted *P* = 1.12 × 10^−7^, OR = 7.70) and MT-TAS2 (adjusted *P* = 3.57 × 10^−4^, OR = 3.62, Fisher’s exact test versus the remainder of the D-loop). Interestingly, these regions are well-defined critical regions at the start and end of mitochondrial transcription. Indeed, the MT-HSP1 and MT-TAS2 are highly conserved regions (Fig. 5A) and alterations in these regions may have profound effects on mitochondrial transcription.

### Validation of shortlisted mutations in the baseline plasma samples

Based on validation selection criteria (Materials and Methods), we shortlisted two mutations: mt.1888G > A and mt.16093T > C. We designed a nested case-control study (Fig. 1) and used ddPCR to quantify the HL of the mtDNA mutations in 663 plasma samples collected before the clinical diagnosis of breast cancer (case: 304, control: 359). According to the distribution of the HL, the study population was dichotomized according to a natural cut-off (HL < 10%: wild type, HL ≥ 10%: mutated, Additional file 2: Fig. S2). The baseline characteristic of the study population is shown in Additional file 2: Table S5. Compared to cancer-free individuals, cases had higher education and higher proportion of hypertension. The baseline characteristics stratified by the mutations show that the presence (proportion) of mt.1888G > A was decreased in individuals with positive family history of breast cancer (Additional file 2: Table S6). There was no statistical difference observed for other clinical variables included in the study. We further performed crude and adjusted logistic regression analysis to investigate the association between mt.1888G > A/mt.16093T > C and breast cancer incidence. Our results show that the presence of mt.16093T > C was significantly associated with an increased risk of breast cancer before (crude OR: 1.64, 95% confidence interval [CI] 1.09–2.20) and after (adjusted OR:1.67, 95% CI 1.11–2.24) adjusting for potential confounding factors (Table 2).

During the follow-up of 304 patients with incident breast cancer, a total of 48 participants died (all-cause mortality), and we performed multivariate cox proportional analysis and investigated the association between cancer mortality and shortlisted mutations. We found that there is no statistically significant association between the presence of the mutations and all-cause as well as cancer-specific mortality (Additional file 2: Table S7, Fig. S4).

**Table 2:** Odd ratios and 95% confidence intervals of cancer incidence associated with the mutations

Mutation	No. of cases	No. of controls	Crude OR (95% CI)	Model 1^a^	Model 2^b^	Model 3^c^	Model 4^d^
mt.1888G > A							
Heteroplasmy level	–	–	0.70 (0.16–1.24)	0.69 (0.15–1.23)	0.68 (0.14–1.23)	0.66 (0.11–1.21)	0.67 (0.12–1.22)
Dichotomized							
Wild type	280	320	1 (Ref)	1 (Ref)	1 (Ref)	1 (Ref)	1 (Ref)
Mutated	24	39	0.70 (0.17–1.24)	0.69 (0.16–1.23)	0.68 (0.14–1.23)	0.66 (0.12–1.21)	0.67 (0.13–1.22)
mt.16093T > C							
Heteroplasmy level	–	–	1.64 (1.06–2.21)	1.63 (1.06–2.21)	1.65 (1.07–2.23)	1.70 (1.12–2.29)	1.68 (1.10–2.27)
Dichotomized							
Wild type	272	335	1 (Ref)	1 (Ref)	1 (Ref)	1 (Ref)	1 (Ref)
Mutated	32	24	1.64 (1.09–2.20)	1.63 (1.08–2.19)	1.65 (1.09–2.21)	1.70 (1.13–2.26)	1.67 (1.11–2.24)

^a^ Adjusted for age, BMI

^b^ Adjusted for age, BMI, education level, smoking habits, alcohol consumption

^c^ Adjusted for age, BMI, education level, smoking habits, alcohol consumption, activity at work, activity at home

^d^ Adjusted for age, BMI, education level, smoking habits, alcohol consumption, activity at work, activity at home, diabetes, hypertension, family history of cancer

## Discussion

In this study, we sequenced breast cancer biopsies and matched baseline cancer-free whole blood samples from the same women in a Swedish population-based cohort, to investigate the mtDNA evolutional mutation landscape in patients with breast cancer. Our results show that breast tumors have a selectively higher mutation load compared to matched whole blood, suggesting that mtDNA mutations, especially heteroplasmic mutations, may contribute to breast cancer pathogenesis. Furthermore, we found significantly higher heteroplasmic mutational load in critical mitochondrial genomic regions in breast cancer biopsies. Finally, we have identified and validated a heteroplasmic mutation -- mt.16093T > C, which was associated with higher incidence of breast cancer. These findings can potentially be used for earlier detection of breast cancer.

The mtDNA exhibits a heightened mutability in contrast to nuclear DNA, which attributed to the cumulative effects of elevated exposure to reactive oxygen species (ROS) and the increased frequency of mtDNA replication. The assessments of the human germline mitochondrial DNA (mtDNA) mutation rate indicate a range of 1.30 × 10^−8^ to 1.89 × 10^−8^ mutations per site per year [31].However, the majority of reported mtDNA mutations associated with human diseases are known to be heteroplasmic and are involved in diseases when mutations accumulate and the HL exceeds a certain threshold, which might depend on the affected tissue, age, and region (nonsynonymous/synonymous/D-loop) of the mitochondrial genome [32]. Evidence has suggested that the accumulation of somatic mtDNA mutations leads to mitochondrial dysfunction and influences cancer progression and metastasis [16]. MtDNA somatic mutations in cancers are abundant, but their selective occurrence exhibits a strong dependency on specific genes and contextual factors. The dN/dS is a quantitative measure of selection with value > 1 indicate positive selection, < 1 indicate negative selection [33]. The mtDNA mutation load was found to be highly lineage-specific, with specific cancer types manifesting a markedly elevated burden of mtDNA mutations when compared to others. Additionally, it became evident that mtDNA serves as a major source of driver mutations in the context of cancer [14].

The roles of mitochondrial mutations have also been previously investigated in breast cancer and important mtDNA mutations have been identified. For example, mutations in the *CYB* gene and D-loop were widely identified to be associated with breast cancer, and mutations in other genes including mt.1811 A > G, mt.5390 A > G, mt.8472 C > A, mt.9966G > A and mt.10,398 A > G were also found to be associated with breast cancer risk [34, 35]. Since the D-loop region is highly susceptible to reactive oxygen species (ROS) damage and due to the absence of extensive mtDNA repair mechanism, most of the mutations accumulate in D-loop [36]. Except for common somatic mutations in the general population, most of the reported breast cancer-associated mutations in D-loop region were found in this study, however, major mutations reported in the *CYB* genes were missing in our study population [35]. The well-studied mt.10,398 A > G mutation, which is presented in the *ND3* gene, can promote oxidation, affect ATP production and contribute to breast cancer development, has been shown and validated in different populations for the association of breast cancer risk [37–41], was also found in our study population. The mt.1888G > A mutation in the *RNR2* has been previously reported in patients with maternally inherited diabetes and deafness [42] and other chronic diseases [43–47], and was one of the haplogroup JT defining mutations. From our sequenced breast cancer biopsies, we found 2.3% of participants belonged to haplogroup JT. However, we detected 8.4% (56 out of 663 in plasma samples) of participants carrying mt.1888G > A mutation and this mutation for the first time was found to be associated with first-degree family history of cancer in our study. The mitochondrial nucleotide position mt.16,093 is a hot spot where different substitutions have been observed in different tissues of the same individual [48]. One previous study reported mt.16093T > C mutation in the blood of patients with breast cancer [49], which is in line with our findings. However, we additionally show that the HL of mt.16093T > C mutation was higher in breast cancer biopsies compared to matched baseline blood samples. Interestingly, we could also detect this mutation in plasma samples collected from the same patient before the clinical diagnosis. We further show that higher HL of the mt.16093T > C in plasma was associated with higher incidence of breast cancer. These results further confirm that it is not just the presence of the mutation but the HL of the mutation which may contribute to cancer. Taken together, we suggest that this mutation has the potential to be a biomarker of early breast cancer diagnosis using a “liquid biopsy” from blood/plasma.

It has been reported that most tumor types show neutral evolution of the mitochondrial genome [50], however, in this study, we demonstrate a positive selection of mtDNA mutation in breast cancer. The breast biopsies harbor 1.17-fold higher proportions of overall nonsynonymous mutations over synonymous mutations and 2.67-fold higher proportions of heteroplasmic nonsynonymous over synonymous mutations. For the germline (homoplasmic) mutation, we also found positive selection of mtDNA mutation. Interestingly, in cases of de novo heteroplasmic mutations, the majority of the genes show positive selection, except *ATP8* and *ND4L* which have no nonsynonymous mutations. Mitochondrion has an evolutionally higher frequency of C to T transitions compared to other mutations. In our study, biopsies had higher C to all other types of bases substitution; we speculate that substitution from C to nucleotides other than T in biopsies could be specific to breast cancer.

The mtDNA D-loop mutations in specific sites are known to influence mtDNA replication and have been associated with the increased incidence of multiple cancers [51–57]. Compared to other regions, D-loop had higher hetero- and homo-plasmic mutation frequency in our study population and higher HL in the breast biopsies than the matched blood samples. Among the D-loop regions, the presence of mutations in the non-conserved MT-TFH, conserved MT-HSP1 and MT-TAS2 sub-regions were significantly associated with breast cancer. MT-TFH is the mitochondrial transcription factor A binding site for the regulation of the initiation and termination of mtDNA transcription. MT-HSP1 is the mitochondrial heavy strand promoter 1 that drives the transcription of the 16S and 12S ribosomal RNA [58]. MT-TAS2 contains a promotor that is responsible for the initiation of mtDNA transcription, a conserved sequence block that binds the mtDNA replication factors and a replication origin that interacts with mtDNA helicases [59]. Considering the importance of these three sub-regions in mtDNA replication and gene expression, mutations in these critical regions are likely to hinder normal mitochondrial function, which may contribute to cancer pathogenesis. Based on our results, further studies investigating the role of mutations in these sub-regions and their function in cancer are warranted.

The main strength of this study is that we have comprehensively characterized the whole mitochondrial genome landscape in biopsies and matched baseline whole blood samples, to explore the evolution of cancer mtDNA mutations and identify the de novo mtDNA variants. In addition, we also used ddPCR which enables us to quantify the absolute copy number of both mutant and wild-type mitochondrial mutations (heteroplasmy) with high sensitivity and reproducibility. Furthermore, validation of the shortlisted mutations by a nested case-control study in individuals from a well-defined Swedish population-based cohort, demonstrates that mitochondrial mutations can be detected in plasma samples (liquid biopsy) before the clinical diagnosis and can be molecular biomarkers of early breast cancer. Identification of somatic mutation in liquid biopsy is advantageous compared to tissue biopsy because it is relatively non-invasive, can be collected multiple times and may provide similar information as biopsy. Furthermore, we applied long-range PCR method, which specifically amplifies mtDNA, and therefore rules out the potential contamination of the nuclear genome. Lastly, we refined and characterized the mutations stringently to make sure that we obtained a high-quality mutation list, which is evident from the detection of previously known mutations and of higher Ti/Tv ratio (> 5) for all mutations across our study population and shows the robustness of our data.

The study has some limitations along with its strengths. First, we did not have enough matched baseline whole blood samples, therefore, we were unable to identify additional de novo mutations in those samples. In addition, we did not have enough statistical power to explore the association between breast cancer and haplogroup and make solid conclusions. We lacked the comprehensive clinicopathological data for our breast cancer cases, it is worth highlighting that given the global and Swedish prevalence of the Luminal A breast cancer subtype [60], it is reasonable to infer that a significant proportion of the breast cancer cases included in our analysis could belong to this particular subtype. Moreover, we must emphasize that it is a hypothesis-generating study in which we have characterized mitochondrial genetic landscape in breast cancer biopsies and in matched cancer-free whole blood samples, followed by ddPCR validation of important de novo mutations. Large population-based studies are required for the comprehensive identification of pivotal breast cancer-associated mtDNA mutations. Furthermore, it is imperative to consider the potential for repeated sampling of tissues or blood from the same patient at various time intervals, as this approach would be ideally suited for the dynamic monitoring of the positive selection of mtDNA heteroplasmic mutations in the context of cancer. In addition, the biological mechanisms of how mtDNA mutations contribute to the pathophysiology of tumorigenesis need further experimental evaluation.

## Conclusions

In this study, we have comprehensively characterized the mtDNA mutation landscapes from breast cancer biopsies. By comparing the mtDNA mutations with matched whole blood samples from the same women, we were able to categorize the mutations as germline, de novo and lost mutations. The heteroplasmic shift shows evidence of positive selection in breast cancer. We shortlisted mutations of interest and validated them in a nested case-control study. The identified mt.16093T > C mutation has the potential to be an early breast cancer diagnosis biomarker using liquid biopsy, which may provide new insights into the development of more accurate and individualized approaches for cancer diagnosis and treatment.

### Supplementary Information

**Additional file 1.** Information of all mtDNA mutations detected in this study after the quanlity control steps.

**Additional file 2: Table S1.** MtDNA mutational characteristics of 86 breast biopsies. **Table S2.** Haplogroup associated disease-causing mutations identified in our study population. **Table S3.** MtDNA mutational characteristics of unmatched 36 breast biopsies. **Table S4.** The dN/dS of de novo heteroplasmic mutations. **Table S5.** Baseline characteristics stratified by incident and no cancers. **Table S6.** Baseline characteristics stratified by shortlisted mtDNA mutations. **Table S7.** Hazard ratios and 95% confidence intervals of mortality associated with mutations among breast cancer patient. **Figure S1.** The mtDNA mutation profiles of 50 matched biopsy-whole blood pairs. **Figure S2.** The alteration probability of mtDNA mutational signature with respect to 96 trinucleotides. **Figure S3.** The HL distribution of mt.1888G > A and mt.16093T > C mutation. **Figure S4.** Kaplan-Meier plot of breast cancer mortality stratified by A. mt.16093T > C and B. mt.1888G > A mutation.

## Data Availability

Restrictions apply to the availability of the raw mtDNA sequencing data and clinical data, which were used under license for the current study, so this part of the supporting data are not publicly available. The all mtDNA variants after the quality control were generated or analyzed during the current study are included in Additional file 1. Further details and analysis codes that support the findings of this study are available from the corresponding author upon request.

## Abbreviations

BRCA1/2: Breast cancer gene 1/2
TP53: Tumor Protein P53
PTEN: Phosphatase And Tensin Homolog
CDH1: Cadherin 1
STK11: Serine/Threonine Kinase 11
CHEK2: Checkpoint Kinase 2
PALB2: Partner And Localizer Of BRCA2
mtDNA: mitochondrial DNA
PCR: Polymerase chain reaction
NGS: Next-generation sequencing
ddPCR: droplet digital PCR
cfDNA: cell-free DNA
HL: heteroplasmy level
CI: Confidence interval
SD: Standard deviation
OR: Odds ratio
QC: Quality control
InDels: Insertions/deletions
