# Supplementary material for: Mitochondrial heteroplasmic shifts reveal a positive selection of breast cancer
Source: J Transl Med. 2023 Oct 5;21:696. doi: 10.1186/s12967-023-04534-4 (PMC10557196; doi:10.1186/s12967-023-04534-4)
Supplement: Supplementary file 2 — Additional file 2: Table S1. MtDNA mutational characteristics of 86 breast biopsies. Table S2. Haplogroup associated disease-causing mutations identified in our study population. Table S3. MtDNA mutational characteristics of unmatched 36 breast biopsies. Table S4. The dN/dS of de novo heteroplasmic mutations. Table S5. Baseline characteristics stratified by incident and no cancers. Table S6. Baseline characteristics stratified by shortlisted mtDNA mutations. Table S7. Hazard ratios and 95% confidence intervals of mortality associated with mutations among breast cancer patient. Figure S1. The mtDNA mutation profiles of 50 matched biopsy-whole blood pairs. Figure S2. The alteration probability of mtDNA mutational signature with respect to 96 trinucleotides. Figure S3. The HL distribution of mt.1888G > A and mt.16093T > C mutation. Figure S4. Kaplan-Meier plot of breast cancer mortality stratified by A. mt.16093T > C and B. mt.1888G > A mutation. [file 12967_2023_4534_MOESM2_ESM.docx]

**Supplementary material**

**Table S1.** MtDNA mutational characteristics of 86 breast biopsies.

**Table S2.** Haplogroup associated disease-causing mutations identified in our study population.

**Table S3.** MtDNA mutational characteristics of unmatched 36 breast biopsies.

**Table S4.** The dN/dS of *de novo* heteroplasmic mutations.

**Table S5.** Baseline characteristics stratified by incident and no cancers.

**Table S6.** Baseline characteristics stratified by shortlisted mtDNA mutations.

**Table S7.** Hazard ratios and 95% confidence intervals of mortality associated with mutations among breast cancer patient.

**Figure S1.** The mtDNA mutation profiles of 50 matched biopsy-whole blood pairs.

**Figure S2.** The alteration probability of mtDNA mutational signature with respect to 96 trinucleotides.

**Figure S3.** The HL distribution of mt.1888G>A and mt.16093T>C mutation.

**Figure S4.** Kaplan-Meier plot of breast cancer mortality stratified by A. mt.16093T>C and B. mt.1888G>A mutation.

**Table S1.** **MtDNA mutational characteristics of 86 breast biopsies**.

|  | Length (bp) | No. of heteroplasmic mutations per sample | No. of homoplasmic mutations per sample | No. of mutations per sample | No. of InDels per sample | Ti/Tv  (Hetero) | Ti/Tv | dN/dS (Hetero) | dN/dS | CpG/Non-CpG  (Hetero) | CpG/Non-CpG |
| --- | --- | --- | --- | --- | --- | --- | --- | --- | --- | --- | --- |
| D-loop | 1122 | 1.42 | 5.45 | 6.87 | 0.36 | 10.89 | 5.63 | - | - | 0.09 | 0.06 |
| Gene | 11341 | 5.50 | 9.28 | 14.78 | 1.01 | 6.25 | 16.29 | 2.67 | 1.17 | 0.11 | 0.11 |
| rRNA | 2513 | 0.86 | 3.21 | 4.07 | 0.78 | 3.06 | 16.45 | - | - | 0.15 | 0.03 |
| tRNA | 1504 | 0.72 | 0.59 | 1.31 | 0.01 | 10 | 27 | - | - | 0.11 | 0.15 |
| Total | 16480 | 8.5 | 18.53 | 27.03 | 2.16 | - | - | - | - | - | - |

Columns are defined as follows: The length is the total number of bases by mitochondrial DNA region. No of hetero- and homo-plasmic mutations/InDels is the total number of hetero- and homo-plasmic mutations divided by 86 participants. Ti/Tv is the ratio of transition-to-transversion change calculated by the number of (heteroplasmic) mutations with A/G or T/C (transition) change divided by the number of (heteroplasmic) mutations with A/C or T/G (transversion) change. dN/dS is the ratio of (heteroplasmic) nonsynonymous to synonymous substitution calculated by the number of (heteroplasmic) nonsynonymous mutations divided by synonymous mutations in the protein-coding region. CpG/Non-CpG is calculated by the number of (heteroplasmic) mutations presented at CpG island divided by the number of (heteroplasmic) mutations presented at Non-CpG sites.

**Table S2. Haplogroup associated disease-causing mutations identified in our study population.**

| **Pos** | **Ref** | **Alt** | **DP** | **HL** | **SampleId** | **Plasmy** | **Gene** | **Region** | **ProteinPos** | **Consequence** | **RefCondon** | **VarCondon** | **RefAA** | **VarAA** | **Haplogroup** | **Patho_score** |
| --- | --- | --- | --- | --- | --- | --- | --- | --- | --- | --- | --- | --- | --- | --- | --- | --- |
| 3243 | A | G | 786 | 0.018 | GL2069-Biopsy | hetero | TRNL1 | tRNA | NA | NA | NA | NA | NA | NA | H | 1 |
| 4136 | A | G | 1799 | 0.99 | ET2146-Biopsy | homo | ND1 | gene | 277 | nonsynonymous | TAC | TGC | Y | C | H | 0.374 |
| 4160 | T | C | 379 | 0.098 | DQ8219-Biopsy | hetero | ND1 | gene | 285 | nonsynonymous | CTC | CCC | L | P | U | 0.853 |
| 9478 | T | A | 652 | 0.029 | HE2526-Biopsy | hetero | COX3 | gene | 91 | nonsynonymous | GTT | GAT | V | D | H | 0.541 |
| 10197 | G | A | 1955 | 0.167 | FD6901-Biopsy | hetero | ND3 | gene | 47 | nonsynonymous | GCC | ACC | A | T | H | 0.594 |
| 12770 | A | G | 687 | 0.019 | GJ6067-Biopsy | hetero | ND5 | gene | 145 | nonsynonymous | GAG | GGG | E | G | HV | 0.763 |
| 12811 | T | C | 2469 | 0.998 | FE6877-Biopsy | homo | ND5 | gene | 159 | nonsynonymous | TAC | CAC | Y | H | H | 0.587 |
| 13637 | A | G | 659 | 0.995 | GL2069-Biopsy | homo | ND5 | gene | 434 | nonsynonymous | CAA | CGA | Q | R | H | 0.61 |

**Table S3.** **MtDNA mutational characteristics of unmatched 36 breast biopsies**.

|  | Length (bp) | No. of heteroplasmic mutations per sample | No. of homoplasmic mutations per sample | No. of mutations per sample | No. of InDels per sample | Ti/Tv  (Hetero) | Ti/Tv | dN/dS (Hetero) | dN/dS | CpG/Non-CpG  (Hetero) | CpG/Non-CpG |
| --- | --- | --- | --- | --- | --- | --- | --- | --- | --- | --- | --- |
| D-loop | 1122 | 1.58 | 5.14 | 6.72 | 0.33 | 12 | 58 | - | - | 0.12 | 0.07 |
| Gene | 11341 | 6.17 | 9.39 | 15.56 | 0.83 | 4.93 | 12.03 | 2.34 | 1.11 | 0.11 | 0.11 |
| rRNA | 2513 | 0.86 | 3.20 | 4.06 | 0.75 | 3.43 | 19.86 | - | - | 0.19 | 0.04 |
| tRNA | 1504 | 0.78 | 0.64 | 1.42 | 0.03 | 8.5 | 20 | - | - | 0.17 | 0.21 |
| Total | 16480 | 9.39 | 18.37 | 27.76 | 1.94 | - | - | - | - | - | - |

Columns are defined as follows: The length is the total number of bases by mitochondrial DNA region. No of hetero- and homo-plasmic mutations/InDels is the total number of hetero- and homo-plasmic mutations divided by 86 participants. Ti/Tv is the ratio of transition-to-transversion change calculated by the number of (heteroplasmic) mutations with A/G or T/C (transition) change divided by the number of (heteroplasmic) mutations with A/C or T/G (transversion) change. dN/dS is the ratio of (heteroplasmic) nonsynonymous to synonymous substitution calculated by the number of (heteroplasmic) nonsynonymous mutations divided by synonymous mutations in the protein-coding region. CpG/Non-CpG is calculated by the number of (heteroplasmic) mutations presented at CpG island divided by the number of (heteroplasmic) mutations presented at Non-CpG sites.

**Table S4. The dN/dS of *de novo* heteroplasmic mutations.**

| **gene** | **nonsynonymous** | **synonymous** | **dN/dS** |
| --- | --- | --- | --- |
| **ATP6** | 15 | 4 | 3.75 |
| **ATP8** | 0 | 1 | 0 |
| **COX1** | 13 | 5 | 2.6 |
| **COX2** | 5 | 3 | 1.66666666666667 |
| **COX3** | 24 | 8 | 3 |
| **CYTB** | 45 | 25 | 1.8 |
| **ND1** | 97 | 20 | 4.85 |
| **ND2** | 8 | 1 | 8 |
| **ND3** | 6 | 3 | 2 |
| **ND4** | 20 | 9 | 2.22222222222222 |
| **ND4L** | 0 | 2 | 0 |
| **ND5** | 36 | 28 | 1.28571428571429 |

**Table S5.** **Baseline characteristics** **stratified by incident and no cancers.**

|  | No cancer  (n=359) | |  | Incident cancer  (n=304) | | *P* value* |
| --- | --- | --- | --- | --- | --- | --- |
|  | **Mean** | **SD** |  | **Mean** | **SD** |  |
| Age | 56.52 | 3.04 |  | 56.71 | 2.97 | 0.41 |
| BMI | 25.48 | 4.05 |  | 25.27 | 3.83 | 0.48 |
|  |  |  |  |  |  |  |
|  | **No.** | **%** |  | **No.** | **%** |  |
| Education level |  | | | | | 0.04 |
| 0-9 | 191 | 53.2 |  | 147 | 48.4 |  |
| 10-11 | 37 | 10.3 |  | 52 | 17.1 |  |
| $\geq$12 | 131 | 36.5 |  | 105 | 34.5 |  |
| Smoking habit |  | | | | | 0.64 |
| Non-smokers | 73 | 20.3 |  | 64 | 21.1 |  |
| Past smokers | 4 | 1.1 |  | 6 | 2.0 |  |
| Current smokers | 282 | 78.6 |  | 234 | 77.0 |  |
| Alcohol habit |  | | | | | 0.44 |
| No consumption | 83 | 23.1 |  | 68 | 22.4 |  |
| <12 g/day | 238 | 66.3 |  | 194 | 63.8 |  |
| $\geq$12 g/day | 38 | 10.6 |  | 42 | 13.8 |  |
| Activity at work |  | | | | | 0.14 |
| Low | 122 | 34.0 |  | 116 | 38.2 |  |
| Moderate | 126 | 35.1 |  | 115 | 37.8 |  |
| High | 111 | 30.9 |  | 73 | 24.0 |  |
| Activity at home |  | | | | | 0.49 |
| Low | 209 | 58.2 |  | 186 | 61.2 |  |
| High | 150 | 41.8 |  | 118 | 38.8 |  |
| Diabetes |  | | | | | 0.27 |
| No | 297 | 82.7 |  | 262 | 86.2 |  |
| Yes | 62 | 17.3 |  | 42 | 13.8 |  |
| Hypertension |  | | | | | 0.02 |
| No | 188 | 52.4 |  | 188 | 61.8 |  |
| Yes | 171 | 47.6 |  | 116 | 38.2 |  |
| Obesity |  | | | | | 0.98 |
| No | 346 | 96.4 |  | 294 | 96.7 |  |
| Yes | 13 | 3.6 |  | 10 | 3.3 |  |
| 1^st^-degree family history of cancer |  | | | | | 0.47 |
| No | 271 | 75.5 |  | 223 | 73.4 |  |
| Yes | 44 | 12.3 |  | 47 | 15.5 |  |
| Unknown | 44 | 12.3 |  | 34 | 11.2 |  |

* Student’s t-tests were performed for continuous variables. Chi-square tests were performed for categorical variables.

**Table S6. Baseline characteristics stratified by shortlisted mtDNA mutations.**

|  | mt.1888G>A | | |  | mt.16093T>C | | |
| --- | --- | --- | --- | --- | --- | --- | --- |
|  | **Wt (n=600)** | **Mut (n=63)** | *P value** |  | **Wt (n=607)** | **Mut (n=56)** | *P* value* |
|  |  |  |  |  |  |  |  |
| Age, mean | 56.64 (3.01) | 56.44 (3.03) | 0.63 |  | 56.62 (3.00) | 56.59 (3.09) | 0.94 |
| BMI, mean | 25.45 (3.01) | 24.74 (3.03) | 0.10 |  | 25.43 (3.96) | 24.89 (3.81) | 0.32 |
|  |  |  |  |  |  |  |  |
| Education level, No. (%) |  |  | 0.33 |  |  |  | 0.73 |
| 0-9 | 306 (51.0) | 32 (50.8) |  |  | 312 (51.4) | 26 (46.4) |  |
| 10-11 | 77 (12.8) | 12 (19.0) |  |  | 80 (13.2) | 9 (16.1) |  |
| $\geq$12 | 217 (36.2) | 19 (30.2) |  |  | 215 (35.4) | 21 (37.5) |  |
| Smoking habit, No. (%) |  |  | 0.69 |  |  |  | 0.81 |
| Non-smokers | 124 (20.7) | 13 (20.6) |  |  | 123 (20.3) | 14 (25.0) |  |
| Past smokers | 10 (1.7) | 0 (0) |  |  | 9 (1.5) | 1 (1.8) |  |
| Current smokers | 466 (77.7) | 50 (79.4) |  |  | 475 (78.3) | 41 (73.2) |  |
| Alcohol habit, No. (%) |  |  | 0.12 |  |  |  | 0.25 |
| No consumption | 138 (23.0) | 13 (20.6) |  |  | 136 (22.4) | 15 (26.8) |  |
| <12 g/day | 385 (64.2) | 47 (74.6) |  |  | 394 (64.9) | 38 (67.9) |  |
| $\geq$12 g/day | 77 (12.8) | 3 (4.8) |  |  | 77 (12.7) | 3 (5.4) |  |
| Activity at work, No. (%) |  |  | 0.54 |  |  |  | 0.30 |
| Low | 215 (35.8) | 23 (36.5) |  |  | 223 (36.7) | 15 (26.8) |  |
| Moderate | 215 (35.8) | 26 (41.3) |  |  | 219 (36.1) | 22 (39.3) |  |
| High | 170 (28.3) | 14 (22.2) |  |  | 165 (27.2) | 19 (33.9) |  |
| Activity at home, No. (%) |  |  | 0.11 |  |  |  | 0.27 |
| Low | 351 (58.5) | 44 (69.8) |  |  | 366 (60.3) | 29 (51.8) |  |
| High | 249 (41.5) | 19 (30.2) |  |  | 241 (39.7) | 27 (48.2) |  |
| Diabetes |  |  | 0.39 |  |  |  | 0.10 |
| No | 503 (83.8) | 56 (88.9) |  |  | 507 (83.5) | 52 (92.9) |  |
| Yes | 97 (16.2) | 7 (11.1) |  |  | 100 (16.5) | 4 (7.10) |  |
| Hypertension |  |  | 0.26 |  |  |  | 0.44 |
| No | 345 (57.5) | 31 (49.2) |  |  | 341 (56.2) | 35 (62.5) |  |
| Yes | 255 (42.5) | 32 (50.8) |  |  | 266 (43.8) | 21 (37.5) |  |
| Obesity |  |  | 0.16 |  |  |  | 0.73 |
| No | 577 (96.2) | 63 (100) |  |  | 585 (96.4) | 55 (98.2) |  |
| Yes | 23 (3.80) | 0 (0) |  |  | 22 (3.60) | 1 (1.80) |  |
| 1^st^-degree family history of cancer |  |  | 0.04 |  |  |  | 0.48 |
| No | 449 (74.8) | 45 (71.4) |  |  | 456 (75.1) | 38 (67.9) |  |
| Yes | 86 (14.3) | 5 (7.9) |  |  | 81 (13.3) | 10 (17.9) |  |
| Unknown | 65 (10.8) | 13 (20.6) |  |  | 70 (11.5) | 8 (14.3) |  |

* Student’s t-tests were performed for continuous variables. Chi-square tests were performed for categorical variables.

**Table S7. Hazard ratios and 95% confidence intervals of mortality associated with mutations among breast cancer patients.**

| **Mutation** | **No. of patient** | **No. of all-cause death** | **No. of cancer-specific death** | **Adjusted all-cause mortality HR (95% CI) *** | **Adjusted cancer-specific mortality HR (95% CI) *** |
| --- | --- | --- | --- | --- | --- |
| **mt.1888G>A** | | | | | |
| Wild type | 237 | 43 | 38 | 1 (Ref) | 1 (Ref) |
| Mutated | 19 | 5 | 4 | 1.10 (0.68-1.52) | 1.19 (0.00-2.45) |
| **mt.16093T>C** | | | | | |
| Wild type | 230 | 42 | 36 | 1 (Ref) | 1 (Ref) |
| Mutated | 26 | 6 | 6 | 1.18 (0.33-2.04) | 1.11(0.72-1.5) |

*Adjusted for age, BMI, education level, smoking habits, alcohol consumption, activity at work, activity at home, diabetes, family history of cancer, hypertension, death from other reasons.

**Figure S1. The mtDNA mutation profiles of 50 matched biopsy-whole blood pairs.** A-B. The mutation rate of mtDNA genomic regions. The vertical axes represent the number of refined heteroplasmic (A) or homoplasmic (B) variants in each gene divided by the number of samples. The darker color is biopsy and the lighter color is the whole blood sample. Genomic regions on the mitochondrial gene are displayed and colored by the type as A. C-D. Comparison of two groups regarding mitochondrial genes with variants, respective p-value obtained with Poisson regression. E. Comparison of hetero- and homo-plasmic mutation frequency in mitochondrial genome regions between two group. F. Comparison of heteroplasmic InDels mutation frequency between two group. G. Histograms of heteroplasmic levels in each mtDNA region with fitted kernel density curves.

**Figure S2.** **The alteration probability of mtDNA mutational signature with respect to 96 trinucleotides.** A-B. Heteroplasmic mutations in (A) biopsies and (B) matched whole blood samples. C-D. Homoplasmic mutations in (C) biopsies and (D) matched whole blood samples.

**Figure S3. The HL distribution of mt.1888G>A and mt.16093T>C mutation.**

**Figure S4. Kaplan-Meier plot of breast cancer mortality stratified by A mt.16093T>C and B. mt.1888G>A mutation.**
